# The impact of the COVID-19 pandemic on primary health care practices and patient management in the Republic of Moldova - results from the PRICOV-19 survey

**DOI:** 10.1186/s12875-023-02116-3

**Published:** 2023-10-25

**Authors:** Giulia Delvento, Ala Curteanu, Cristina Rotaru, Esther Van Poel, Sara Willems, Helen Prytherch, Ghenadie Curocichin

**Affiliations:** 1 Swiss Tropical and Public Health Institute, P.O. Box, CH-4002, Basel, Switzerland; 2 University of Basel, P.O. Box, CH-4003, Basel, Switzerland; 3 Healthy Life project: Reducing the Burden of Non-Communicable Diseases in Moldova, Chișinău, Moldova; 4 Swiss Agency for Development and Cooperation (SDC), Chișinău, Moldova; 5 Mother and Child Institute, Chișinău, Republic of Moldova; 6 Nicolae Testemitanu Medical University, Chișinău, Republic of Moldova; 7 Department of Public Health and Primary Care, Ghent University, Ghent, Belgium; 8 Quality and Safety Ghent, Department of Public Health and Primary Care, Ghent University, Ghent, Belgium

**Keywords:** COVID-19, Primary health care, Health systems, Republic of Moldova, Family doctor, General practitioner, Infectious disease, PRICOV-19, Quality of care

## Abstract

**Background:**

The COVID-19 pandemic has had an enormous impact on health systems in Europe and has generated unprecedented challenges for tertiary care. Less is known about the effects on the activities of local family doctors (FDs), who have shifted tasks and adapted their practice to accommodate the new services brought by the pandemic. The PRICOV-19 study was a multi-country survey aiming to understand the challenges posed by the pandemic in primary health care (PHC) practices around Europe. Within the framework of this study, we assessed the impact of the pandemic on PHC facilities in urban, rural, and mixed urban/rural areas in the Republic of Moldova.

**Methods:**

We present the results from the PRICOV-19 questionnaire designed at Ghent University (Belgium) and distributed between January and March 2021 to PHC facilities from the 35 districts of the Republic of Moldova. This analysis presents descriptive data on limitations to service delivery, staff role changes, implementation and acceptance of COVID-19 guidelines, and incidents reported on staff and patient safety during the pandemic.

**Results:**

Results highlighted the differences between facilities located in urban, rural, and mixed areas in several dimensions of PHC. Nearly half of the surveyed facilities experienced limitations in the building or infrastructure when delivering services during the pandemic. 95% of respondents reported an increase in time spent giving information to patients by phone, and 88% reported an increase in responsibilities. Few practices reported errors in clinical assessments, though a slightly higher number of incidents were reported in urban areas. Half of the respondents reported difficulties delivering routine care to patients with chronic conditions and a delay in treatment-seeking.

**Conclusions:**

During the pandemic, the workload of PHC staff saw a significant increase, and practices met important structural and organizational limitations. Consequently, these limitations may have also affected care delivery for vulnerable patients with chronic conditions. Adjustments and bottlenecks need to be addressed, considering the different needs of PHC facilities in urban, rural, and mixed areas.

## Introduction

The COVID-19 pandemic has affected healthcare delivery worldwide through unprecedented levels of hospitalizations and subtracting health workers from routine care activities and other medical emergencies. These disruptions may have led to a delay of surgery and diagnoses of other severe diseases, which could worsen the outcomes or prognoses of patients affected by other non-COVID conditions [1, 2]. Studies have shown that the pandemic also had an impact on the activities of primary healthcare (PHC) practices, with a decrease of up to 25% in PHC service use during lockdowns or changes in functions of the practices such as a shift towards serving as the first point of triage for suspect COVID-19 patients [3–6]. These changes were also prompted by new COVID-19 guidelines for general PHC practices implemented in several countries, which recommended, for instance, a postponement of all non-urgent visits and procedures and a shift from face-to-face to video or phone consultations [7, 8].

### The primary healthcare system in Moldova

**Fig. 1:**
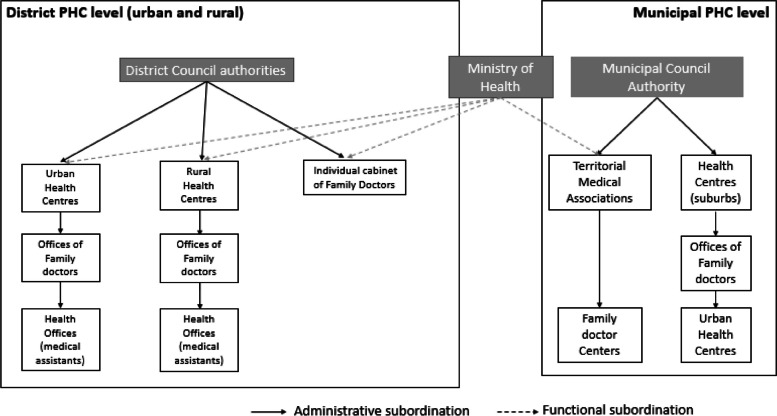
Structure of the primary healthcare system at district and municipal level in the Republic of Moldova

The Republic of Moldova has undergone reforms of its healthcare system, following the dissolution of the Soviet Union and declaration of its independence. Structural and organizational change of health services started in 1991 to move away from the previous Semashko system [9]. The PHC sector reform started in 1997 with the aim to establish a decentralised PHC system based on a Family Medicine model. The reform has gradually led to a transfer of financial resources from tertiary to PHC [10]. Since 2008, the PHC institutions have been recognized as autonomous practices, operating independently from hospitals [10]. At the last stage of reform, the free practice model was introduced to expand access to family doctors (FDs) in rural areas (Governmental Decision n. 988 from 10.10.2018). However, the coverage of family doctors still remains suboptimal nowadays in these areas. A major reform of health care financing started with the introduction of mandatory social health insurance in 2004 [11]. PHC accounts for 35% of the public health expenditure provided by the National Health Insurance Company (NHIC). Public institutions of PHC as well as private ones are contracted by the NHIC. Currently, PHC services in 35 districts of Moldova are delivered through a network of urban and rural health centres, offices of family doctors and health offices and are governed by district councils (see Fig. 1).

In municipalities, Territorial Medical Associations (TMA) oversee and support the management of urban health centres and are comprised of by diagnostic consultative units and FD centres, being governed by district or municipal councils. Generally, FDs operate as a part of PHC teams, with one FD and 2–3 nurses. The Ministry of Health (MoH) provides guidelines and administrative directives for PHC facilities.

In Moldova, the functions of FDs include being the first point of contact between the patient and health system, gate-keeping, providing medical assistance for emergencies, monitoring chronic diseases, supervising of pregnant women and children, as well as following-up child development, prescribing medical treatment and devices, referring patients to a specialized practices or hospital care. According to data from the WHO 2012 Evaluation of Primary Health Care, workload differed between rural and urban areas with rural areas working an average of 45.9 h per week, with a slightly lower average in urban areas of 42.3 h/week [12, 13].

### Management of the COVID-19 pandemic in the Republic of Moldova

**Fig. 2:**
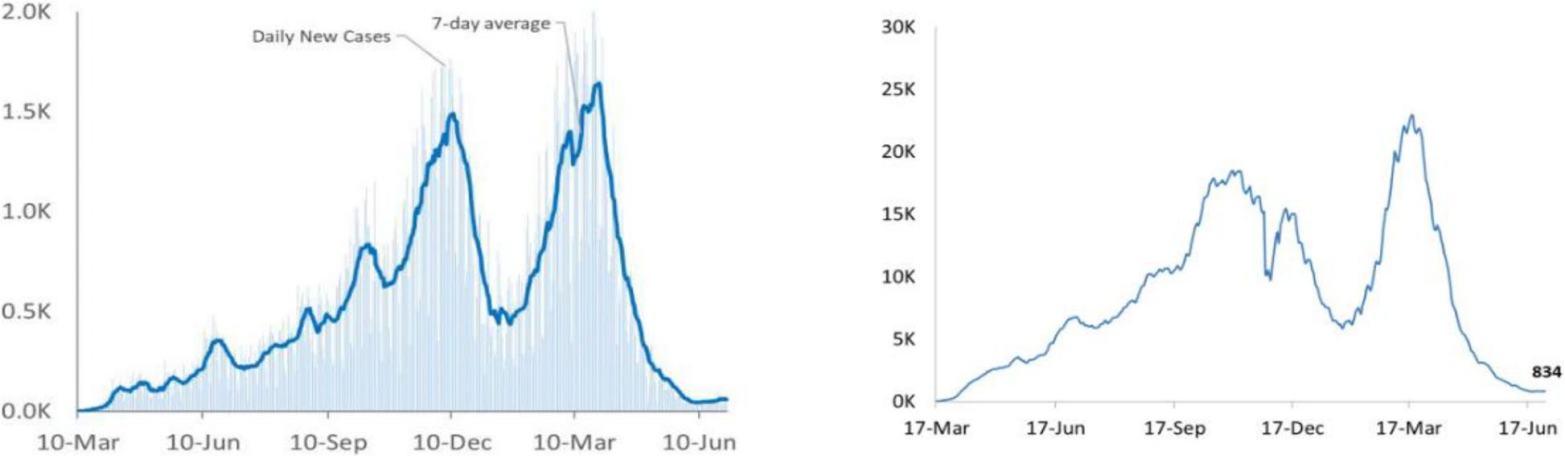
Incidence of COVID-19 in Moldova in March 2021-June 2021 (left) and number of active cases in the same period (right). Source: COVID-19 response and recovery monthly bulletin june 2021-Republic of Moldova

The Republic of Moldova detected its first case of COVID-19 in March 2020 and has had three epidemic waves as of January 2022 (see Fig. 2) [14, 15] similarly to other European countries [16, 17]. In February–March 2020 the MoH implemented different measures to control the spread of COVID-19 and ensure the continuation of health services [18]. To accelerate efforts in the response, a team of national experts from the MoH, in collaboration with WHO, developed and disseminated guidelines for PHC practices, such as the following: a) National clinical protocol for COVID-19; b) the standardized clinical protocol for family doctor for the assessment of COVID-19 patients (updated in March, June, September and December 2020) [19]; c) the practical guideline for the management of severe complications of the coronavirus infection; and d) the national guidelines for the rehabilitation of patients affected by COVID-19 [20]. Furthermore, the MoH also issued guidelines regarding the use of personal protective equipment (PPE) and waste disposal for PHC facilities [21]. The WHO Country office for the Republic of Moldova also provided the training of medical staff on the mentioned protocols across all health sectors. Moreover, new guidelines on remote consultations were developed from a standardized international protocol and disseminated to PHC facilities to ensure the safety of patients and health workers [22]. This guideline reports best practices for conducting these consultations to monitor patients with COVID-19 and other conditions, to prevent the need of home visits and ensure that patients with chronic conditions receive appropriate care. Phone or video consultations were also required for the management and monitoring of mild and moderate cases of COVID-19 by FDs in Moldova. Additionally, the PHC providers had to transmit data on infected patients to the territorial Public Health Department. During the pandemic, rapid antigen tests were introduced in PHC practices in March 2021 and were integrated in PHC activities and delivered through the deployment of mobile teams, travelling to households of patients with suspected COVID-19, or by setting specific testing walk-in hours within the practices [23]. PHCs also carried out health surveillance measures such as registering daily temperature of COVID-19 patients, assessing clinical symptoms of persons coming from areas with high-incidence of COVID-19 and monitoring patients’ contacts by phone for 14 days [24].

Within this context, PHC facilities of the Republic of Moldova took part in the PRICOV-19 survey, a multi-country study aiming to study the impact of the pandemic on PHC practices on the dimensions of quality of care [25]. The study was coordinated by Ghent University (Belgium) and conducted in 37 European countries and Israel to assess how primary care practices were organized during the COVID-19 pandemic, to ensure the maintenance of high-quality services. Our paper aims to investigate how the pandemic affected PHC practices within rural, mixed urban–rural and urban facilities in the Republic of Moldova on different outcomes which can be broadly grouped in the following dimensions: a) Access to appointments and telemedicine services; b) Implementation and perception of COVID-19 guidelines; c) Staff roles and collaboration between PHC practices; and d) Patient safety.

## Methods

### Study design

The PRICOV-19 study was a cross-sectional study rolled out between November 2020 and December 2021 across 38 countries. The questionnaire was developed at Ghent University in multiple phases and aimed to sample over 50 practices per country. The questionnaire was designed based on information collected through a scoping review and Delphi procedure conducted with 5 PHC experts who validated the items and questionnaire length, and was piloted among a group of 159 PHC doctors in Belgium. The end date of data collection was pre-established by the PRICOV consortium so that data collection periods would allow comparability of results between countries. More details are discussed in the study protocol.

In the Republic of Moldova, data was collected between January and March 2021 when the country was amidst its second wave of the pandemic from February to April 2021.

#### Study setting and description of materials

The study involved all 35 districts in the country and the two municipalities of Chișinău and Balti. The area covers a population of approximately 2,597,107 inhabitants [26]. The questionnaire was self-administered electronically to staff working in three types of public facilities, namely urban facilities, rural health centres and offices of family doctors and the participation was voluntary.

The questionnaire was sent out in Romanian and Russian languages to all 293 public PHC facilities in the country through a dedicated channel of the Department of Policy in PHC, emergency and community care of the MoH. Additionally, three reminders via email were sent to increase response rates. Per FD practice, one questionnaire was completed, by a FD or staff familiar with the practice organization. The study protocol and data handling protocols are described in the Data Management Plan registered at Ghent University. The questionnaire consists of 53 items divided into six topics: (a) infection prevention; (b) patient flow for COVID- and non-COVID care; (c) dealing with new knowledge and protocols; (d) communication with patients; (e) collaboration; (f) wellbeing of the respondent; (g) and characteristics of the respondent and practice. The Research Electronic Data Capture (REDCap) platform was used to host the questionnaire in all languages and to securely store the answers from the participant. More details are described elsewhere [10].

### Outcomes

For this analysis, we considered a subset of the questionnaire containing background information on characteristics of facilities and the patient flow for COVID and non-COVID care.

To understand access to PHC services and whether practices were prepared beforehand to receive possible COVID-19 patients, we assessed several factors. Namely, the implementation of phone and video consultations and whether PHC staff were collecting information from the patients regarding the reasons for making appointments.We additionally assessed whether the practices had walk-in hours and still implemented home visits, as well as the presence of limitations to infrastructure of the facilities. We also investigated the implementation and acceptance of guidelines and recommended practices such as disinfection, by assessing the frequency of use and whether the COVID-19 guidelines posed a threat to the safety of personnel and organization of the PHC practice. Furthermore, we assessed changes in staff roles, satisfaction of change and shifts in team collaboration and external collaboration with other practices. Lastly incidents during patient management and delays in providing care were also investigated.

To compare outcomes, we generated a composite variable which merged location (urban, rural and mixed) and type of facility (health centres and offices of FDs) to consider contextual differences in the organization of the PHCs in these geographical areas. We also assessed differences in outcomes by number of patients registered per FD, subdividing it into quartiles, for the sections on incidents, as we expected to observe differences owing to the higher or lower workloads for health professionals.

### Statistical analyses

Ghent University was responsible for the preliminary data cleaning of the international data, while further cleaning and analysis were performed by the local team at the Nicolae Testemitanu State Medical University in Chișinău and by partners at the Swiss Tropical and Public Health Institute. We established a cut-off point for inclusion in analysis of the observations with 50% of valid responses to the analysed sections. The dataset contained both scale data and categorical and Likert-type data. To increase power, multiple-choice and Likert-type responses were recoded to binary or fewer categories for some variables. Missing values were considered in both tabulations and tests and sensitivity analysis was conducted to verify if the exclusion of missing values had changed the outcomes.

We described absolute and relative frequencies and performed chi2 or Fisher’s test on categorical variables to test for associations between variables and used the ANOVA method on scale data. The criterion of statistical significance (two-fold, p) is determined at 0.05. A descriptive methodology was selected to describe relative and absolute frequencies and explore relations between variables. The small sample size hindered the use of multivariable regression to explore associations between specific outcomes and multiple predictors.

## Results

### Characteristics of facilities

Among 293 PHC facilities that received the invitation to participate, the questionnaire was accessed 148 times of which 81/148 (53%) of attempts were excluded because of having more than 50% of missing values within the data subset. 67/148 respondents (43%) completed the responses of interest for the analysis. 25/67 (37.3%) of respondents had between 20 and 29 years of experience, and 14/67 (21%) had less than 9 years and these were predominantly located in rural areas. 53/67 (79%) were FDs and 9/67 (13%) were facility managers and 5/67 (8%) responded as a team. Facilities servicing only rural population had a lower number of FDs with an average of 3.2 FDs working per facility vs. 57 FDs per facility serving large urban areas. Urban areas had also a greater number of patients with 68,348 patients registered or population covered on average vs. 6,376 patients registered in rural facility given that urban facilities have a larger capacity and a more complex structure than mixed/rural facilities. 14 out of 44 rural facilities reported more than 2,260 patients per FD and all urban facilities falling below this category (see Table 1).

**Table 1:** Health Provider (respondent) characteristics by location (urban, rural and mixed) and type of facility

**Provider characteristics**	**Urban**	**Mixed**	**Rural**	**Total**	
	***N* = 10**	***N* = 13**	***N* = 44**	***N* = 67**	***P* Value**
	n (%)	n (%)	n (%)	n (%)	
**Years of experience of respondent**					
0–9	0 (0)	2 (15.4)	12 (27.3)	14 (20.9)	0.06
10–19	1 (10)	2 (15.4)	12 (27.3)	15 (22.4)	
20–29	6 (60)	7 (53.9)	12 (27.3)	25 (37.3)	
More than 30 years	3 (30)	1 (7.7)	2 (4.6)	6 (9)	
Missing	0 (0)	1 (7.7)	6 (13.6)	7 (10.5)	
**Profession/type of respondent**					
FD	7 (70)	9 (69.2)	37 (84.1)	53 (79.1)	0.17
Manager	3 (30)	3 (23.1)	3 (6.8)	9 (13.4)	
Team	0 (0)	1 (7.7)	4 (9.1)	5 (7.5)	
**N. of staff**					
Mean n. of FDs + FD trainees	57.2	14.3	3.2	13.6	0.000
SD	44.5	7.01	2.46	25.5	
Mean n. of FTEs	63.6	23	4.8	16.6	0.000
SD	52.8	13.2	5.3	3.64	
**Payment of staff**					
Mean n. of paid Staff	318.8	125.4	24.6	84.5	0.000
SD	270.2	54.8	27.7	17.6	
**Population covered or registered in facility**					
Mean n. of patients registered or population served	68,348	27,005	6,378	19,611	0.000
SD	18,732.8	3117.8	860.5	388.3	
**Population served by 1 FD**					
Mean n. of Patients per FD	1,449	2,013	2,051	1,952	0.000
SD	197.6	178.1	112.1	89.3	
**Quartiles of n. of patients per FD**					
Q1 < 1533	5 (50)	2 (15.4)	9 (20.5)	16 (23.9)	0.287
Q2 1533–1835	3 (30)	4 (30.8)	10 (22.7)	17 (25.4)	
Q3 1835–2260	2 (20)	4 (30.8)	11 (25)	17 (25.4)	
Q4 > 2260	0 (0)	3 (23.1)	14 (31.8)	17 (25.4)	

### Limitations, appointments and telemedicine

**Fig. 3:**
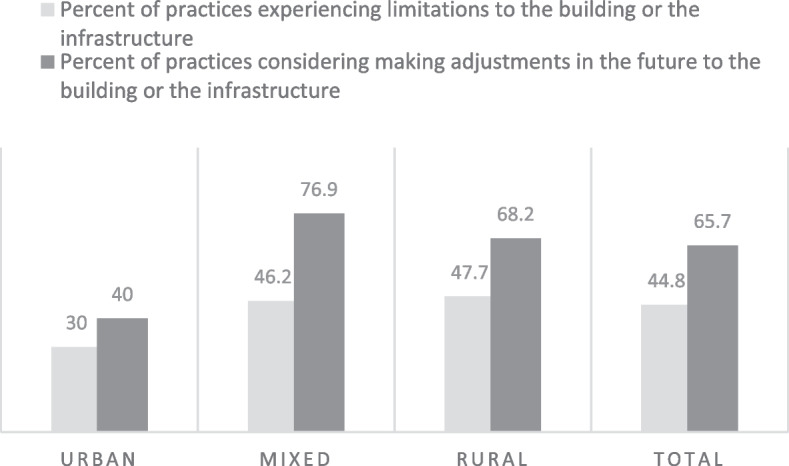
Percent of primary healthcare facilities experiencing limitations and making adjustments to their practice during the COVID-19 pandemic in rural, urban and mixed areas

Overall, 35/67 (52%) of respondents reported that they did not experience any / hardly any limitations to the building or infrastructure during the pandemic, while 30/67 (45%) responded they experienced limitations to a limited or large extent (see Table 1 and Fig. 3). 44/67 (65%) of respondents reported that the pandemic led the practice to consider future adjustments to the building with rural and mixed areas requiring slightly more adjustments than in urban areas (more than 68% of facilities in rural and mixed areas require adjustments vs. 40% in urban areas).

We found that 58/67 facilities (86%) complied with asking for reasons for making an appointment by phone. 80% of urban practices and more than 90% of rural and mixed practices had walk-in hours for appointments and 51 out of 67 respondents (75%) reported that home visits were organized in a way that COVID-19 patients are seen at the end of the round of the FD.

**Fig. 4:**
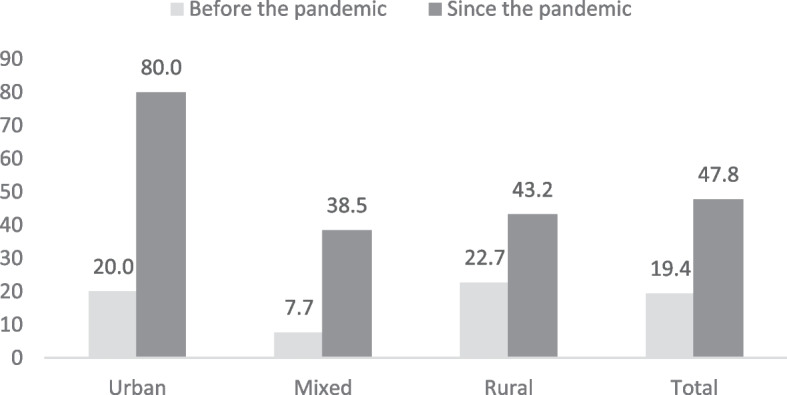
Percent of primary healthcare facilities using video consultations before and during the pandemic in urban, rural and mixed areas

When investigating the availability and functioning of an online appointment system25 out of 67 responses were non-applicable or missing (37%), especially from rural areas (20/44, 46%), indicating that online appointments may have not been implemented. Before the pandemic, only 23% of urban and rural facilities and 1/13 (7%) of mixed facilities used video consultations. Since the pandemic, there has been an increase up to 32/67 (47%) of facilities using video consultations, with the highest increase seen in urban areas, which showed an increase from 2/10 (20%) to 8/10 (80%) of facilities using video consultations (see Table 2 and Fig. 4).

**Table 2:** Limitations to implementing appointments and telemedicine practices (*p < 0.05)

Appointments and routine activities		Urban	Mixed	Rural	Total
		***N* = 10**	***N* = 13**	***N* = 44**	***N* = 67**
		n (%)	n (%)	n (%)	n (%)
**Limitations related to the building or the infrastructure of this practice to provide high-quality and safe care**	None or Hardly	7 (70)	7 (53.9)	21 (47.7)	35 (52.2)
	To a limited or large extent	3 (30)	6 (46.2)	21 (47.7)	30 (44.8)
	NA	0 (0)	0 (0)	2 (4.6)	2 (3)
**Did the COVID-19 pandemic lead this practice to consider making adjustments in the future to the building or the infrastructure?**	None or Hardly	6 (60)	3 (23.1)	14 (31.8)	23 (34.3)
	To a limited or large extent	4 (40)	10 (76.9)	30 (68.2)	44 (65.7)
**The practice has walk-in hours for consultations**	Yes	8 (80)	13 (100)	40 (90.9)	61 (91)
**The online appointment system informs patients about which complaints they may (not) bring to the practice**	Yes	4 (40)	4 (30.8)	14 (31.8)	22 (32.8)
**Patients must state a reason when making an online appointment**	Yes	3 (30)	8 (61.5)	16 (36.4)	27 (40.3)
**Patients must state a reason when making a phone appointment**	Yes	9 (90)	13 (100)	36 (81.8)	58 (86.6)
**Home visits are organized so that potential COVID-19 patients are seen at the end of the round of the GP**	Never/rarely	0 (0)	1 (7.7)	4 (9.1)	5 (7.5)
	Sometimes	0 (0)	1 (7.7)	0 (0)	1 (1.5)
	Regularly	2 (20)	5 (38.5)	18 (40.9)	25 (37.3)
	Always	5 (50)	4 (30.8)	17 (38.6)	26 (38.8)
	NA/missing	3 (30)	2 (15.4)	5 (11.4)	10 (14.9)
**Did the practice use video consultations BEFORE the COVID-19 pandemic?**	No, never	7 (70)	12 (92.3)	33 (75)	52 (77.6)
	Yes	2 (20)	1 (7.7)	10 (22.7)	13 (19.4)
	Missing	1 (10)	0 (0)	1 (2.3)	2 (3)
**Did the practice use video consultations SINCE the COVID-19 pandemic?**	No, never	1 (10)	8 (61.5)	23 (52.3)	32 (47.8)
	Yes	8 (80)	5 (38.5)	19 (43.2)	32 (47.8)
	Missing	1 (10)	0 (0)	2 (4.6)	3 (4.5)

### Implementation and perception of guidelines and protocols

The phone protocol for the management of COVID-19 patients was available in 65/67 (97%) of facilities, and information on how to refer a patient to a triage station was available in 85% of facilities. In 45 out of 67 (67%) facilities, phone consultation protocols were being used always, and 18/67 (27%) reported regular use. 51/67 (76%) of respondents reported that if non-FD staff performed the phone triages, these health workers can always rely on FDs support.

Half of respondents reported that they received adequate support from the government for the proper functioning of the practice, 23/67 (34%) were neutral to this statement and 7/67 (10%) of respondents reported that they did not receive adequate support. Thirty-six out of 67 (54%) of respondents replied that did not feel a threat to their well-being due to the MoH guidelines though 5/10 (40%) of urban facilities did feel a threat compared to 2/13 (16%) of those in mixed and 12/44 (27%) of the respondents in rural areas. A minority responded that they felt that the guidelines affected the good functioning of their organization (16%). The pandemic had also required an increased need for disinfection of indoor premises and we found a high compliance of 61/67 (91%) of facilities reporting that they could consistently disinfect consultation rooms in-between visits (see Table 3).

**Table 3:** Implementation of IPC measures, phone triage, and perception of guidelines, protocols and government support in PHC practices

Implementation and perception of guidelines		Urban	Mixed	Rural	Total
		***N* = 10**	***N* = 13**	***N* = 44**	***N* = 67**
		n (%)	n (%)	n (%)	n (%)
**Sufficient time is provided between consultations for the disinfection of the consultation room**	Never/rarely	1 (10)	0 (0)	2 (4.6)	3 (4.5)
	Sometimes	0 (0)	0 (0)	3 (6.8)	3 (4.5)
	Regularly	2 (20)	4 (30.8)	11 (25)	17 (25.4)
	Always	7 (70)	9 (69.2)	27 (61.4)	43 (64.2)
	NA/missing	0 (0)	0 (0)	1 (2.3)	1 (1.5)
**The guidelines imposed by the government on PC practices as a consequence of COVID-19 pose a threat to the good organization of this practice**	Disagree/Strongly dis	5 (50)	7 (53.9)	26 (59.1)	38 (56.7)
	Neutral	3 (30)	1 (7.7)	11 (25)	15 (22.4)
	Agree/strongly agree	2 (20)	3 (23.1)	6 (13.6)	11 (16.4)
	DK/Missing	0 (0)	2 (15.4)	1 (2.3)	3 (4.5)
**The guidelines imposed by the government on PC practices as a consequence of COVID-19 pose a threat to the personal well-being of the staff in this practice**	Disagree/Strongly dis	5 (50)	8 (61.5)	23 (52.3)	36 (53.7)
	Neutral	1 (10)	1 (7.7)	7 (15.9)	9 (13.4)
	Agree/strongly agree	4 (40)	2 (15.4)	12 (27.3)	18 (26.9)
	DK/Missing	0 (0)	2 (15.4)	2 (4.6)	4 (6)
**Adequate support is provided by the government for the proper functioning of this practice**	Disagree/Strongly dis	1 (10)	1 (7.7)	5 (11.4)	7 (10.5)
	Neutral	3 (30)	4 (30.8)	16 (36.4)	23 (34.3)
	Agree/strongly agree	6 (60)	6 (46.2)	22 (50)	34 (50.8)
	DK/Missing	0 (0)	2 (15.4)	1 (2.3)	3 (4.5)
**A protocol is used practice when answering phone calls from potential COVID-19 patients**	Yes	10 (100)	12 (92.3)	43 (97.7)	65 (97)
**When answering these phone calls, how often is this protocol used in this practice?**	Sometimes	0 (0)	0 (0)	1 (2.3)	1 (1.5)
	Regularly	2 (20)	5 (38.5)	11 (25)	18 (26.9)
	Always	8 (80)	7 (53.9)	30 (68.2)	45 (67.2)
	NA/missing	0 (0)	1 (7.7)	2 (4.6)	3 (4.5)
**If phone triage is performed by non-GP staff, he/she can rely on support from a GP if needed**	Sometimes	0 (0)	0 (0)	2 (4.6)	2 (3)
	Regularly	3 (30)	3 (23.1)	7 (15.9)	13 (19.4)
	Always	7 (70)	10 (76.9)	34 (77.3)	51 (76.1)
	NA/missing	0 (0)	0 (0)	1 (2.3)	1 (1.5)
**Information on how to refer a patient to a triage station is available**	Yes	10 (100)	10 (76.9)	37 (84.1)	57 (85.1)

### Staff roles and collaboration

95% of facilities reported increased times spent giving information by phone and giving information to patients with low health literacy. This activity, involved all types of respondents, including facility managers (88%). More than 90% of respondents also reported increased involvement in triage (63/67) and actively reaching out to fragile patients who need to be followed up (61/67). 88% of staff responded that their responsibilities increased during this period, though 25/67 (37%) reported being happy about task shifting, Eighteen out of 67 (27%) reported neutrality and 17/67 (25%) were not happy about it. 6/10 (60%) of urban facilities reported higher satisfaction levels with task shifting compared to 4/13 (31%) of respondents in mixed and 15/44 (34%) rural facilities. Furthermore, half of the respondents confirmed needing further training for these amended responsibilities.

There are also differences between respondents in different areas regarding their perception of being prepared towards task shifting, with 6/10 (60%) of respondents in urban facilities feeling prepared for these new tasks compared to 7/13 (53%) and 16/44 (36%) of respondents feeling prepared in mixed and rural facilities. When assessing differences by roles of the respondents, managers felt more prepared compared to FDs (7/9, 77% vs 22/53, 41%).

We also assessed how work was handled in case of absences of staff, and overall 48/67 (71%) of practices responded that work was re-distributed among colleagues. However, there were slight differences among facilities, with 9/10 (90%) of urban facilities responding that there is a re-distribution of tasks vs. 7/13 (53%) in mixed and 32/44 (72%) in rural facilities. Urban facilities fair better also in case of attrition. Six out of 10 60% Urban facilities report always having another colleague to whom to transfer administrative and medical files compared to 5/13 (38%) of mixed and 21/44 (47%) of rural facilities. Additionally, 36/67 (54%) of practices reported that they could rely on the help of other practices in the area in case of absences of colleagues. Overall 38/67 (57%) reported an improvement in collaboration with other practices during the pandemic, 8/67 (12%) were neutral and 15/67 (22%) reported that no improvement in cooperation was perceived (see Table 4).

**Table 4:** Changes in staff roles, staff satisfaction and collaboration (*p < 0.05)

Staff roles & collaboration		Urban	Mixed	Rural	Total	GP	Manager	Team	Total
		***N* = 10**	***N* = 13**	***N* = 44**	***N* = 67**	***N* = 53**	***N* = 9**	***N* = 5**	***N* = 67**
		n (%)	n (%)	n (%)	n (%)	n (%)	n (%)	n (%)	n (%)
**Staff members are more involved in giving information to patients by phone**	Disagree/Strongly disagree	0 (0)	0 (0)	1 (2.3)	1 (1.5)	0 (0)	1 (11.1)	0 (0)	1 (1.5)
	Neutral	0 (0)	1 (7.7)	0 (0)	1 (1.5)	1 (1.9)	0 (0)	0 (0)	1 (1.5)
	Agree/strongly agree	10 (100)	12 (92.3)	42 (95.5)	64 (95.5)	51 (96.2)	8 (88.9)	5 (100)	64 (95.5)
	Missing	0 (0)	0 (0)	1 (2.3)	1 (1.5)	1 (1.9)	0 (0)	0 (0)	1 (1.5)
**Staff members are more involved in giving information to illeterate/low health literacy or migrants**	Disagree/Strongly disagree	0 (0)	1 (7.7)	2 (4.6)	3 (4.5)	0.2 (3.8)	1 (11.1)	0 (0)	3 (4.5)
	Neutral	0 (0)	0 (0)	2 (4.6)	2 (3)	2 (3.8)	0 (0)	0 (0)	2 (3)
	Agree/strongly agree	10 (100)	12 (92.3)	39 (88.6)	61 (91)	48 (90.6)	8 (88.9)	5 (100)	61 (91)
	Missing	0 (0)	0 (0)	1 (2.3)	1 (1.5)	1 (1.9)	0 (0)	0 (0)	1 (1.5)
**Staff members are more involved in actively reaching out to patients that might postpone healthcare**	Disagree/Strongly disagree	0 (0)	0 (0)	1 (2.3)	1 (1.5)	1 (1.9)	0 (0)	0 (0)	1 (1.5)
	Neutral	0 (0)	1 (7.7)	2 (4.6)	3 (4.5)	2 (3.8)	1 (11.1)	0 (0)	3 (4.5)
	Agree/strongly agree	9 (90)	12 (92.3)	40 (90.9)	61 (91)	48 (90.6)	8 (88.9)	5 (100)	61 (91)
	Missing	1 (10)	0 (0)	1 (2.3)	2 (3)	2 (3.8)	0 (0)	0 (0)	2 (3)
**Staff members are more involved in the triage of patients**	Disagree/Strongly disagree	0 (0)	1 (7.7)	0 (0)	1 (1.5)	1 (1.9)	0 (0)	0 (0)	1 (1.5)
	Neutral	0 (0)	1 (7.7)	1 (2.3)	2 (3)	2 (3.8)	0 (0)	0 (0)	2 (3)
	Agree/strongly agree	10 (100)	11 (84.6)	42 (95.5)	63 (94)	49 (92.5)	9 (100)	5 (100)	63 (94)
	Missing	0 (0)	0 (0)	1 (2.3)	1 (1.5)	1 (1.9)	0 (0)	0 (0)	1 (1.5)
**GPs or GP trainees are more involved in actively reaching out to patients that might postpone healthcare**	Disagree/Strongly disagree	1 (10)	0 (0)	2 (4.6)	3 (4.5)	0.2 (3.8)	1 (11.1)	0 (0)	3 (4.5)
	Neutral	0 (0)	1 (7.7)	4 (9.1)	5 (7.5)	5 (9.4)	0 (0)	0 (0)	5 (7.5)
	Agree/strongly agree	9 (90)	11 (84.6)	36 (81.8)	56 (83.6)	43 (81.1)	8 (88.9)	5 (100)	56 (83.6)
	Don't know/Missing	0 (0)	1 (7.7)	2 (4.6)	3 (4.5)	3 (5.7)	0 (0)	0 (0)	3 (4.5)
**My responsibilities in this practice increased**	Disagree/Strongly disagree	0 (0)	1 (7.7)	0 (0)	1 (1.5)	1 (1.9)	0 (0)	0 (0)	1 (1.5)
	Agree/strongly agree	10 (100)	11 (84.6)	38 (86.4)	59 (88.1)	50 (94.3)	9 (10)	0 (0)	59 (88.1)
	Missing	0 (0)	1 (7.7)	6 (13.6)	7 (10.5)	2 (3.8)	0 (0)	5 (100)	7 (10.5)
**I am happy with the task shifting in my professional role since the COVID-19 pandemic**	Disagree/Strongly disagree	2 (20)	4 (30.8)	11 (25)	17 (25.4)	15 (28.3)	2 (22.2)	0 (0)	17 (25.4)
	Neutral	2 (20)	4 (30.8)	12 (27.3)	18 (26.9)	16 (30.2)	2 (22.2)	0 (0)	18 (26.9)
	Agree/strongly agree	6 (60)	4 (30.8)	15 (34.1)	25 (37.3)	20 (37.7)	5 (55.6)	0 (0)	25 (37.3)
	Missing	0 (0)	1 (7.7)	6 (13.6)	7 (10.5)	2 (3.8)	0 (0)	5 (100)	7 (10.5)
**I do not feel prepared for the task shifting in my professional role since the COVID-19 pandemic**	Disagree/Strongly disagree	6 (60)	7 (53.9)	16 (36.4)	29 (43.3)	22 (41.5)	7 (77.8)	0 (0)	29 (43.3)
	Neutral	1 (10)	4 (30.8)	13 (29.6)	18 (26.9)	17 (32.1)	1 (11.1)	0 (0)	18 (26.9)
	Agree/strongly agree	3 (30)	1 (7.7)	9 (20.5)	13 (19.4)	12 (22.6)	1 (11.1)	0 (0)	13 (19.4)
	Missing	0 (0)	1 (7.7)	6 (13.6)	7 (10.5)	2 (3.8)	0 (0)	5 (100)	7 (10.5)
**I need further training for these amended responsibilities since the COVID-19 pandemic**	Disagree/Strongly dis	2 (20)	4 (30.8)	11 (25)	17 (25.4)	15 (28.3)	2 (22.2)	0 (0)	17 (25.4)
	Neutral	2 (20)	1 (7.7)	5 (11.4)	8 (11.9)	7 (13.2)	1 (11.1)	0 (0)	8 (11.9)
	Agree/strongly agree	6 (60)	6 (46.2)	22 (50)	34 (50.8)	28 (52.8)	6 (66.7)	0 (0)	34 (50.8)
	Don't know/Missing	0 (0)	2 (15.4)	6 (13.6)	8 (11.9)	3 (5.7)	0 (0)	5 (100)	8 (11.9)
**If staff members leave this practice, is there a transfer to another colleague of the files that need follow-up? This can be both administrative and medical records**	Never/rarely	0 (0)	1 (7.7)	4 (9.1)	5 (7.5)	3 (5.7)	2 (22.2)	0 (0)	5 (7.5)
	Sometimes	0 (0)	0 (0)	1 (2.3)	1 (1.5)	0 (0)	0 (0)	1 (20)	1 (1.5)
	Usually	4 (40)	5 (38.5)	12 (27.3)	21 (31.3)	18 (34)	2 (22.2)	1 (20)	21 (31.3)
	Always	6 (60)	5 (38.5)	21 (47.7)	32 (47.8)	26 (49.1)	4 (44.4)	2 (40)	32 (47.8)
	NA/Missing	0 (0)	2 (15.4)	1 (13.6)	3 (11.9)	6 (11.3)	1 (11.1)	1 (20)	8 (11.9)
**If staff members in this practice are absent because of COVID-19 (infection or quarantine), the work can be distributed in such a way that the well-being of colleagues is not compromised**	Disagree/Strongly dis	0 (0)	2 (15.4)	5 (11.4)	7 (10.5)	6 (11.3)	1 (11.1)	0 (0)	7 (10.5)
	Neutral	1 (10)	1 (7.7)	5 (11.4)	7 (10.5)	6 (11.3)	1 (11.1)	0 (0)	7 (10.5)
	Agree/strongly agree	9 (90)	7 (53.9)	32 (72.7)	48 (71.6)	37 (69.8)	6 (66.7)	5 (100)	48 (71.6)
	DK/Missing	0 (0)	3 (23.1)	2 (4.6)	5 (7.5)	4 (7.6)	1 (11.1)	0 (0)	5 (7.5)
**If staff members in this practice are absent because of COVID-19 (infection or quarantine), this practice can count on the help of other PC practices in the neighborhood**	Disagree/Strongly dis	1 (10)	3 (23.1)	11 (25)	15 (22.4)	10 (18.9)	4 (44.4)	1 (20)	15 (22.4)
	Neutral	2 (20)	2 (15.4)	6 (13.6)	10 (14.9)	9 (17)	1 (11.1)	0 (0)	10 (14.9)
	Agree/strongly agree	7 (70)	4 (30.8)	25 (56.8)	36 (53.7)	29 (54.7)	3 (33.3)	4 (80)	36 (53.7)
	DK/Missing	0 (0)	4 (30.8)	2 (4.6)	6 (9)	5 (9.4)	1 (11.1)	0 (0)	6 (9)
**The COVID-19 pandemic has promoted cooperation with other PC practices in the neighborhood**	Disagree/Strongly dis	3 (30)	2 (15.4)	10 (22.7)	15 (22.4)	13 (24.5)	2 (22.2)	0 (0)	15 (22.4)
	Neutral	1 (10)	1 (7.7)	6 (13.6)	8 (11.9)	7 (13.2)	1 (11.1)	0 (0)	8 (11.9)
	Agree/strongly agree	6 (60)	7 (53.9)	25 (56.8)	38 (56.7)	29 (54.7)	5 (55.6)	4 (80)	38 (56.7)
	DK/Missing	0 (0)	3 (23.1)	3 (6.8)	6 (9)	4 (7.6)	1 (11.1)	1 (20)	6 (9)

### Patient safety management

Out of 67 respondents, 22 (33%) reported that a patient with a non-COVID febrile condition was seen late due to the implementation of the COVID-19 protocol which delayed the care (see Table 4). Delay in seeing a patient was also reported due to the patient delaying seeking care in 9/13 (69%) mixed facilities, 20/44 (46%) of rural facilities and 3/10 (30%) of urban facilities (p = 0.012). Sixteen percent of facilities also reported that a patient was seen late because s/he did not know how to call on a FD. Incorrect assessments of non-COVID patients were infrequent and reported in 6/67 (9%) of facilities overall, with more urban facilities reporting these incidents (20%) compared to the average. Ninety-four percent of respondents reported also that meetings were held if incidents about quality of care occurred.

45/67 (67%) of facilities reported checking whether a patient is able to complete referral to another facility and 63/67 (93%) report checking whether patients are able to isolate themselves in case of infection (see Table 5).

**Table 5:** Practices in patient safety management (*p < 0.05)

Patient safety management		Urban	Mixed	Rural	Total	Population served by 1 Gp				
		***N* = 10**	***N* = 13**	***N* = 44**	***N* = 67**	**Q1 < 1533** ***N* = 16**	**Q2 1534–1835** ***N* = 17**	**Q3 1836–2260** ***N* = 17**	**Q4 > 2260** ***N* = 17**	**Total**
		n (%)	n (%)	n (%)	n (%)	n (%)	n (%)	n (%)	n (%)	n (%)
**When a patient is referred to another facility e.g. the hospital, the triage station,… it is checked whether he/she is able to go there**	**Never**	0 (0)	0 (0)	1 (2.3)	1 (1.5)	0 (0)	0 (0)	0 (0)	1 (5.9)	1 (1.5)
	**Regularly**	4 (40)	5 (38.5)	12 (27.3)	21 (31.3)	4 (25)	8 (47.1)	4 (23.5)	5 (29.4)	21 (31.3)
	**Always**	6 (60)	8 (61.5)	31 (70.5)	45 (67.2)	12 (75)	9 (52.9)	13 (76.5)	11 (64.7)	45 (67.2)
**When a patient needs to isolate him/herself, the extent to which this is feasible at his/her home is checked with the patient**	**Never/rarely**	0 (0)	0 (0)	1 (2.3)	1 (1.5)	0 (0)	0 (0)	0 (0)	1 (5.9)	1 (1.5)
	**Sometimes**	0 (0)	1 (7.7)	2 (4.6)	3 (4.5)	0 (0)	0 (0)	2 (11.8)	1 (5.9)	3 (4.5)
	**Regularly**	5 (50)	5 (38.5)	11 (25)	21 (31.3)	6 (37.5)	6 (35.3)	3 (17.7)	6 (35.3)	21 (31.3)
	**Always**	5 (50)	7 (53.9)	30 (68.2)	42 (62.7)	10 (10)	11 (11)	12 (12)	9 (9)	42 (42)
**A patient with a fever caused by an infection other than COVID-19 was seen late due to the fact the COVID-19 protocol was followed which delayed the care**		3 (30)	6 (46.2)	13 (29.6)	22 (32.8)	5 (31.3)	6 (35.3)	4 (23.5)	7 (41.2)	22 (32.8)
A patient with an urgent condition was seen late because he/she delayed seeking treatment*		3 (30)	9 (69.2)	20 (45.5)	32 (47.8)	4 (25)	8 (47.1)	10 (58.8)	10 (58.8)	32 (47.8)
**A patient with a serious condition was seen late because he/she did not know how to call on a GP**		2 (20)	2 (15.4)	7 (15.9)	11 (16.4)	3 (18.8)	2 (11.8)	3 (17.7)	3 (17.7)	11 (16.4)
**A patient with an urgent condition was seen late because the situation was assessed as non-urgent during the telephonic triage**		1 (10)	1 (7.7)	3 (6.8)	5 (7.5)	1 (6.3)	1 (5.9)	2 (11.8)	1 (5.9)	5 (7.5)
**A patient with an urgent condition other than COVID-19 was assessed incorrectly during the triage procedure**		2 (20)	2 (15.4)	2 (4.6)	6 (9)	1 (6.3)	2 (11.8)	3 (17.7)	0 (0)	6 (9)

## Discussion

The study highlighted different strengths and weaknesses of the PHC system in Moldova during the pandemic, and the differences existing between facilities in different areas. The majority of respondents from facilities serving mixed and urban populations had more than 20 years of experience. This is reflecting findings from other studies showing that 48% of FDs working in Moldova are in (or close to) retirement age [27]. This did not impact though the uptake of technological improvements which were seen concerning video consultation practices as a clear increase in the use of video consultations was observed in all locations from 13/67 (19%) of PHCs using this means before the pandemic to 32/67 (47%) of usage.. In-person services such as walk-in consultations and home visits were maintained in the majority of facilities (61/67, 91% and 51/67, 75%, respectively). These high rates of in-person interaction may also be attributed to the use of PHCs as centres to collect COVID-19 specimens for PCR testing. While ad hoc testing centres were set up in the urban areas, specimen collection for testing in rural areas were provided through home visits by mobile teams or walk-in hours at PHCs [27].

Nearly half of PHC facilities experienced limitations in terms of building or infrastructure due to the pandemic (45%) and more than half reported the need for future adjustments to the facility. This need could be attributed to the fact that many rural facilities were set-up in houses or other types of premises that were not specifically designed as health facilities. In a multi-country analysis of the PRICOV data assessing infrastructural limitations among 33 European countries involved in the study (including Moldova), the percentage of facilities experiencing limitations was 58% across the entire sample [28]. Additionally, the association between experiencing infrastructural limitations and other factors associated with the PHC practice characteristics were explored. Namely, infrastructural limitations were associated with not having sufficient infection prevention control measures in place in the practice (1.38 (1.05; 1.83) *p* = 0.023), the number of GPs (having 2–5 GPs per practice vs. solo practices OR 1.53 (1.27; 1.85) *p* < 0.001) and type of payment system in place in the PHC practice (a fee-for service payment system was associated with lower odds of experiencing limitations compared to capitation: OR 0.73 (0.57; 0.94) *p* = 0.014) [28].

The availability of online appointment systems remains unclear due to the high number of non-applicable responses for these questions (42%). In Moldova, there is an Electronic Health Information System in in place for PHCs, with an integrated online appointment system though, according to other studies this system is not functional in all districts, especially in rural areas due to a lack of adequate IT equipment, internet connection and digital capacities [10, 29, 30].

Other qualitative findings reported that rural PHCs were not equipped with proper areas for collection of samples and storage separated from the rest of the facility, nor was waste disposal equipment available, indicating a further inadequacy of these facilities for collecting samples [31].

There has been an overall increase in workload of the staff and since the beginning of the pandemic, during which, 88% of respondents reported having an increase in responsibilities compared to before the pandemic and nearly all staff spent more time on the phone giving information to patients. Fifty-two percent of respondents reported neutrality or dissatisfaction toward the amended responsibilities. This could be also associated to the high burden of mild and moderate cases to be managed by FDs via remote consultations as shown in another study. In fact, the daily number of people on home treatment between October 2020 and March 2021 ranged from 4000 to 9000 [18].

Another multi-country publication of the PRICOV study assessing distress and well-being of GPs from 33 countries, showed that Moldova, together with Romania, Italy and Bosnia and Herzegovina were the countries with highest scores of well-being (eWBI score of more than 4). Factors associated with having higher well-being were larger sizes of the practice, perceiving of being adequately supported by the government, and experiencing more collaboration with other practices. However the self-reporting method selected and volunteer bias are important limitations that also need to be taken into consideration when interpreting these results [32].

When asked about preparedness regarding task shifting, half of respondents also expressed a need for further training across all types of PHC practices, though we did not assess in what topic the training was most needed. Since the beginning of the pandemic, 44 training sessions were organized and delivered by MoH in collaboration with different UN organizations. The training was centred on COVID-19 prevention and treatment and organized online. Some issues were pointed out in a qualitative evaluation of PHC practices in which FDs reported the need of refresher training and a need to enforce rules regarding the wearing of PPE by medical staff [27].

Ninety-five percent of respondents reported having meetings to discuss with colleagues when incidents happened in the practice. In case of absences of personnel, health workers could re-distribute the work with other colleagues, though urban facilities fared better than rural and mixed ones (9/10, 90% report appropriate substitution of staff vs. 7/13, 53% in mixed and 32/44 72% in rural facilities). Furthermore, a great majority of respondents referred that non-FD staff could always count on the support of a FD for triaging.

There was a higher percentage of facilities reporting incidents due to staffs’ incorrect clinical assessments in urban areas. However, the majority of incidents were owing to delays in treatment seeking on behalf of the patient which delayed administering appropriate care (32/67, 47%).This issue was reported more frequently in mixed and rural areas compared to urban facilities. Another common reason for not seeing a patient on time was attributed to complying with the COVID-19 protocol (22/67, 32%), though the reasons explaining this issue were not collected. The urban location of the facilities may have also had the highest burden of COVID-19 patients as shown in another study conducted in Moldova at the beginning of the pandemic in which 91% hospitalized for severe COVID were coming from urban areas and 9% from rural areas [33].

The pandemic also brought improvements in quality of care such as a better quality of cancer care through the modification of the outpatient consultation pathway. From treatment being accessed only at specialized care centers, the consultation pathway now entails the use of phone consultations for prescription drugs, and, if appropriate, a referral to local PHC providers before accessing specialized care. This has cut waiting times for receiving care and increased the number of providers receiving training on prescription drugs for palliative and antineoplastic drugs [34].

### Limitations

There were several limitations in this study. Firstly, the small sample size did not allow to identify statistically significant results when comparing study groups. Secondly, the facilities that participated may not be representative of all facilities operating in Moldova and thirdly these facilities may also not be most affected by the pandemic. Furthermore, the survey relied on self-reported information. The low response rate and exclusion of observations due to missing data are also a major limitation impacting the representativeness of study results. Health staff having higher workloads and that may have been more affected by the pandemic, may have not responded to the questionnaire due to lack of time, therefore results may be biased due to data submitted by facilities which faired better during this pandemic.

## Conclusion

Results showed that, despite the improvements in primary care achieved in the past years in Moldova during the COVID-19 pandemic, staffing levels of PHCs remains sub-optimal especially in rural and mixed areas with a high proportion of FDs with more than 20 years of experience. The under-staffing of the workforce must be urgently addressed to guarantee the provision of essential PHC services in Moldova. Moreover, shortages of FDs and medical assistants have been reported especially in rural areas which may be attributable to a chronic outmigration towards urban settings and efforts should be made to increase incentives for FDs to guarantee services in rural areas. Respondents have also expressed the need for adjustments to the building and infrastructure of the facilities, though more data is needed to understand the extent and nature of this issue, especially in rural areas where the limitations were more frequently reported.

The increase in time spent of staff in handing out information to patients during the pandemic, also points out a need for a more structured and well-functioning health information system for PHCs. PHC practices in Moldova are also burdened with a high prevalence of chronic diseases, especially cardiovascular diseases which need follow-up care [5]. Services should be guaranteed even in times of the pandemic so that patients can avoid delaying treatment for NCDs and have access to hospitals, with a particular attention to patients located in mixed and rural facilities.

Urban area had the highest burden of COVID-19 patients and this may also explain the higher number of erroneous assessments of patients reported in urban facilities (20% vs. the average 9%). In conclusion, as identified in previous studies [10], the centralized PHC health system in Moldova made several steps to ensure service continuity during the pandemic, by maintaing both in-person consultations updating patient case management and practice guidelines. However, some areas of improvement can be identified, for instance, in the need to address the long-existing disparities between rural and urban facilities, in terms of the structural limitations, and the differences in patient per doctor ratio which translate into a higher workload for rural health facilities. It is also important to ensure in all facilities, proper replacement of personnel in case of absence or attrition, to understand the safety needs, to increase the use of remote consultations and to improve the follow-up of chronically ill patients that need continuous care even during the pandemic.

## Data Availability

The data is available upon reasonable request.

## Abbreviations

FD: Family Doctor
NHIC: National Health Insurance Company
PHC: Primary Health Care
MoH: Ministry of Health
TMA: Territorial Medical Association

## References

[1] Mansfield KE, Mathur R, Tazare J, Henderson AD, Mulick AR, Carreira H (2021). Indirect acute effects of the COVID-19 pandemic on physical and mental health in the UK: a population-based study. Lancet Digital Health.

[2] Maringe C, Spicer J, Morris M, Purushotham A, Nolte E, Sullivan R (2020). The impact of the COVID-19 pandemic on cancer deaths due to delays in diagnosis in England, UK: a national, population-based, modelling study. Lancet Oncol.

[3] Mughal F, Mallen CD, McKee M. The impact of COVID-19 on primary care in Europe. The Lancet Regional Health – Europe. 2021;6. 10.1016/j.lanepe.2021.100152 PMC8245160 34226894

[4] The Health Foundation. Exploring the fall in A&E visits during the pandemic London, UK2020 [cited 2021. Available from: https://www.health.org.uk/news-and-comment/charts-and-infographics/exploring-the-fall-in-a-e-visits-during-the-pandemic.

[5] Park S, Elliott J, Berlin A, Hamer-Hunt J, Haines A. Strengthening the UK primary care response to covid-19. BMJ. 2020;370:m3691. 10.1136/bmj.m3691. Accessed Apr 2022. 10.1136/bmj.m3691 32978177

[6] Order of Approval of Nomenclature of Primary Care Practices in the Republic of Moldova. In: Ministry of health lasp, editor. Chişinău2021.

[7] Tsopra R, Frappe P, Streit S, Neves AL, Honkoop PJ, Espinosa-Gonzalez AB (2021). Reorganisation of GP surgeries during the COVID-19 outbreak: analysis of guidelines from 15 countries. BMC Fam Pract.

[8] Rawaf S, Allen LN, Stigler FL, Kringos D, Quezada Yamamoto H, van Weel C (2020). Lessons on the COVID-19 pandemic, for and by primary care professionals worldwide. Eur J Gen Pract.

[9] World Health Organization Regional Office for Europe (2012). Republic of Moldova: health system review.

[10] Boerma W, Snoeijs S, Wiegers T, Baltag V. Evaluation of the structure and provision of primary care in the Republic of Moldova: a survey-based project. 2012.

[11] Maclehose L, McKee M, Organization WH. Health care systems in transition: Republic of Moldova. 2002.

[12] World Health Organization. Evaluation of the structure and provision of primary care in Moldova: a survey-based project. 2012.

[13] Hone T, Habicht J, Domente S, Atun R (2016). Expansion of health insurance in Moldova and associated improvements in access and reductions in direct payments. J Glob Health.

[14] Osipov T, Malic A, Niguleanu A, Lesnic E, Paladi C. The epidemiological and clinical characteristic of COVID-19 cases from the Republic of Moldova. Eur Respiratory J. 2021;58(suppl 65):PA3269.

[15] Zach H, Hanová M, Letkovičová M (2021). Distribution of COVID-19 cases and deaths in Europe during the first 12 peak weeks of outbreak. Cent Eur J Public Health.

[16] James N, Menzies M, Radchenko P (2021). COVID-19 second wave mortality in Europe and the United States. Chaos.

[17] Cinaglia P, Cannataro M. Forecasting COVID-19 Epidemic Trends by Combining a Neural Network with R(t) Estimation. Entropy (Basel). 2022;24(7). 10.3390/e24070929 PMC9322732 35885152

[18] Council of Euope. COVID-19 in the Republic of Moldova: moving ahead. Available from: https://www.coe.int/en/web/democracy-and-human-dignity/covid-19-newsroom/-/asset_publisher/ueOjQLU2N7mp/content/covid-19-in-the-republic-of-moldova-moving-ahead/14181903. Accessed Sep 2023.

[19] Ministry of Health of the Republic of Moldova. Ministerial order n. 1181 On the approval of the Standardized Clinical Protocol for Family Doctors "Coronavirus infection of new type (COVID-19)", edition IV. Chisinau, Republic of Moldova2020.

[20] Ministry of Health of the Republic of Moldova. Country COVID-19 Intra-action Review of the Republic of Moldova. 2020.

[21] Ministry of Health Labour and Social protection. Ministerial order n. 253 on the approval of the implementation of measures for the prevention and control of infection for the novel Coronavirus (COVID-19) in primary health care. 2020.

[22] Greenhalgh T, Koh GCH, Car J. Covid-19: a remote assessment in primary care. BMJ. 2020;368:m1182. 10.1136/bmj.m1182. 10.1136/bmj.m1182 32213507

[23] Ministry of Health of the Republic of Moldova. Ministerial order n. 169 On the use of rapid diagnostic tests for the detection of Ag SARS-CoV-2. Chisinau, Republic of Moldova2021.

[24] Extraordinary National and Territorial Public Health Commission MoHLaSP. Preparedeness and response plan for the novel Coronavirus disease of the Republic of Moldova. Chisinau, Republic of Moldova; 2020 March 13, 2020. Contract No.: Version 3.

[25] Van Poel E, Vanden Bussche P, Klemenc-Ketis Z, Willems S (2022). How did general practices organize care during the COVID-19 pandemic: the protocol of the cross-sectional PRICOV-19 study in 38 countries. BMC Primary Care.

[26] Statistics Nof. Moldova National database, Population Statistics by sex years 2014–2021 Chisinau, Republic of Moldova 2022 [Available from: https://statbank.statistica.md/PxWeb/pxweb/ro/20%20Populatia%20si%20procesele%20demografice/20%20Populatia%20si%20procesele%20demografice__POPrec__POP010/POP010100rcl.px/?rxid=b2ff27d7-0b96-43c9-934b-42e1a2a9a774. Accessed Sep 2023.

[27] Lozan O, RG, Mihai Pisla M, Ceban A. Report on process evaluation of the organisation and coordination of Health Services in the Republic of Moldova during COVID-19. Fundația Soros Moldova.; 2020. Available from: https://soros.md/publicatii/raport-evaluarea-procesului-de-organizare-si-coordonare-a-serviciilor-de-sanatate-din-republica-moldova-in-conditiile-epidemiei-covid-19/. Accessed Apr 2022.

[28] Windak A, Nessler K, Van Poel E, Collins C, Wójtowicz E, Murauskiene L, et al. Responding to COVID-19: the suitability of primary care infrastructure in 33 countries. Int J Environ Res Public Health. 2022;19(24):17015. 10.3390/ijerph192417015. 10.3390/ijerph192417015 PMC9779330 36554901

[29] Blake C, Bohle LF, Rotaru C, Zarbailov N, Sava V, Sécula F, et al. Quality of care for non-communicable diseases in the Republic of Moldova: a survey across primary health care facilities and pharmacies. BMC Health Serv Res. 2019;19:353. 10.1186/s12913-019-4180-4. 10.1186/s12913-019-4180-4 PMC6547568 31164125

[30] Ministry of Health Republic of Moldova. Analysis of the Health System Development Strategy for the period 2008–2017 in the Republic of Moldova. 2018.

[31] CIVIS- Centre for socio-political analysis WHO. Perceptions, attitudes and behaviour of health professionals in the context of the COVID-19 pandemic 2020.

[32] Collins C, Clays E, Van Poel E, Cholewa J, Tripkovic K, Nessler K, et al. Distress and Wellbeing among General Practitioners in 33 Countries during COVID-19: Results from the Cross-Sectional PRICOV-19 Study to Inform Health System Interventions. International Journal of Environmental Research and Public Health. 2022;19(9). 10.3390/ijerph19095675 PMC9101443 35565070

[33] Ungurean A, Malic A, Osipov T, Lesnic E (2021). The peculiarities of patients with COVID-19 infection. The Moldovan Medical Journal.

[34] World Health Organization. Improving cancer care pathways to address the COVID-19 pandemic: experiences from the Republic of Moldova. Copenhagen: World Health Organization. Regional Office for Europe; 2020. Contract No.: WHO/EURO:2020–1356–41106–55850.

